# Synergistic Effect of Transmyocardial Revascularization and Platelet-Rich Plasma on Improving Cardiac Function After Coronary Artery Bypass Grafting

**DOI:** 10.7759/cureus.60254

**Published:** 2024-05-14

**Authors:** Zain Khalpey, Ujjawal Kumar, Usman Aslam, Jessa Deckwa, John Konhilas

**Affiliations:** 1 Department of Cardiothoracic Surgery, HonorHealth, Scottsdale, USA; 2 School of Clinical Medicine, University of Cambridge, Cambridge, GBR; 3 Department of General Surgery, HonorHealth, Phoenix, USA; 4 Department of Research, Nihon Kohden Digital Health Solutions, Irvine, USA; 5 Department of Research, NFSci, LLC, Tucson, USA

**Keywords:** coronary artery disease (cad), speckle tracking echocardiography (ste), platelet-rich plasma (prp), transmyocardial revascularization (tmr), coronary artery bypass grafting (cabg)

## Abstract

Background

Coronary artery disease (CAD) is a global health burden, contributing to mortality and morbidity. A proportion of patients with CAD suffer from diffuse CAD, where conventional revascularization techniques such as percutaneous coronary intervention and coronary artery bypass grafting (CABG) may be insufficient to adequately restore myocardial perfusion. Transmyocardial revascularization (TMR) uses a laser to create microscopic channels in the myocardium, inducing inflammation, angiogenesis, and neovascularization to improve perfusion to ischemic regions. Platelet-rich plasma (PRP) is an autologous concentrate of platelets that contains a myriad of growth factors and bioactive proteins, which have been shown to promote tissue regeneration and wound healing. The combination of TMR and PRP therapies has been proposed to synergistically enhance myocardial revascularization and functional recovery in patients with advanced CAD undergoing surgical revascularization.

Methods

This study evaluated the efficacy of combining TMR and PRP with CABG in improving cardiac function in diffuse CAD patients. Fifty-two patients were randomized to CABG alone (n = 16), CABG+TMR (n = 17), CABG+PRP (n = 10), and CABG+TMR+PRP (n = 9). TMR was performed using a holmium:YAG laser to create 10 channels in the inferolateral left ventricular wall. PRP was prepared from autologous whole blood and injected into the myocardium adjacent to the TMR channels. Cardiac function was assessed using speckle-tracking echocardiography preoperatively, postoperatively, and at one-year follow-up. Adverse events, including post-operative atrial fibrillation, acute kidney injury, and readmissions, were also recorded. Statistical analyses were performed to compare outcomes between the treatment groups.

Results

The CABG+TMR+PRP group showed significantly improved global longitudinal strain (GLS) at one year compared to CABG alone (mean GLS -15.96 vs -12.09, p = 0.02). Post-operative left ventricular ejection fraction trended higher in the TMR+PRP group (57.78%) vs other groups, but not significantly. Post-operative atrial fibrillation was higher in the TMR+PRP group (67% vs 25%, p = 0.04), potentially reflecting increased inflammation. No significant differences were observed in other adverse events.

Conclusions

The results of this study suggest a synergistic benefit of combining TMR and PRP therapies as an adjunct to CABG in patients with diffuse CAD. The significant improvement in GLS at one year in the TMR+PRP group compared to CABG alone indicates enhanced myocardial remodeling and functional recovery, which may translate to improved long-term outcomes. The higher incidence of postoperative atrial fibrillation in the TMR+PRP group warrants further investigation but may reflect the heightened inflammatory response necessary for angiogenesis and tissue regeneration. Prospective, randomized controlled trials with larger sample sizes and longer follow-up periods are needed to validate these findings and optimize treatment protocols. Nonetheless, concomitant TMR+PRP therapy represents a promising approach to augmenting myocardial revascularization and recovery in patients with advanced CAD undergoing surgical revascularization.

## Introduction

Coronary artery disease (CAD) caused by atherosclerosis may trigger an ischemic event, typically a myocardial infarction (MI). CAD impacts 154 million people worldwide and represents 32.7% of cardiovascular disease [1,2]. The standard treatment options include percutaneous coronary intervention (PCI) or coronary bypass graft surgery (CABG) to revascularize the under-perfused myocardial tissue. However, there are a significant number of end-stage diffuse CAD patients, approximately 10% of all CAD patients, who undergo these procedures and have refractory angina and incomplete vascularization due to diffusely diseased vasculature [3-5].

A solution that has been proposed for recurrent angina and diffuse CAD is the use of transmyocardial revascularization (TMR) as an independent therapy or as an adjunct to CABG [6]. TMR is an invasive cardiac procedure that involves the use of CO_2_ or holmium:YAG laser to create dozens of micropores (1-mm) transmural channels from the epicardial layer to the endocardial surface [7]. This procedure induces inflammation, endothelialization, and neovascularization in myocardial tissues to renew vascular supply in ischemic areas of the myocardium, thereby improving the function of heart tissue that would not be able to be revascularized with a traditional CABG [8-10]. There has been significant evidence in animal models that TMR results in significantly improved myocardial perfusion [11].

Some long-term studies have found that TMR can improve regional wall motion, ejection fraction (EF), contractility, and overall heart function and increase overall survival [12,13]. Many studies have also reported improvement in the reduction of angina symptoms compared to control groups [14,15]. When used as an adjunctive therapy to CABG, there is improved 30-day mortality, decreased need for further revascularization, and decreased medication and inotropic support [15,16]. However, other studies have reported no improvement in heart perfusion with stress testing in comparison to medical therapy alone [17]. Further studies by this group also showed decreased measures of ischemia in patients receiving TMR versus medical therapy controls. The difference in outcomes and impact on cardiac function with the use of TMR in the literature warrants further investigation with more sensitive measurements.

The use of TMR as an adjunct to CABG is also being explored to include the use of mesenchymal stem cells (MSCs) as well as platelet-rich plasma (PRP). The lab clinical studies reported that MSCs could be beneficial in CABGs by enhancing the cardiac repairing process by enhancing neovascularization and neomyogenesis while also reducing tissue fibrosis [18]. Clinical studies have reported that this adjunctive therapy in CABG can enhance myocardial changes and lead to improved ejection fraction and more favorable symptomatology [19,20]. PRP has been used in the past for CABG to reduce the rate of graft failures and sternal wound complications because of platelet growth factors and anti-inflammatory and anti-microbial effects [21,22]. PRP has also been shown to increase stem cell growth (VEGF) and differentiation [23]. With these identified benefits, PRP has been proposed to enhance the endothelialization and neovascularization induced by TMR because of its synergistic effect to induce angiogenesis [24,25].

In the present study, we evaluate and discuss the effectiveness of TMR combined with PRP to improve cardiac function synergistically in patients undergoing CABG. We seek to show this through the evaluation of echocardiograms and speckle-tracking echocardiography (STE) to measure global longitudinal strain (GLS) and left ventricular ejection fraction (LVEF).

## Materials and methods

A single-center, single-surgeon retrospective analysis of CABG patients was performed between 2019 and 2021. Fifty-two patients were enrolled in the study and randomized to four experimental groups: CABG only (n = 16), CABG/TMR (n = 17), CABG/PRP (n = 10), and CABG/TMR/PRP (n = 9). All subjects in this study also received concomitant left atrial appendage ligation.

Operative procedure

**Figure 1:**
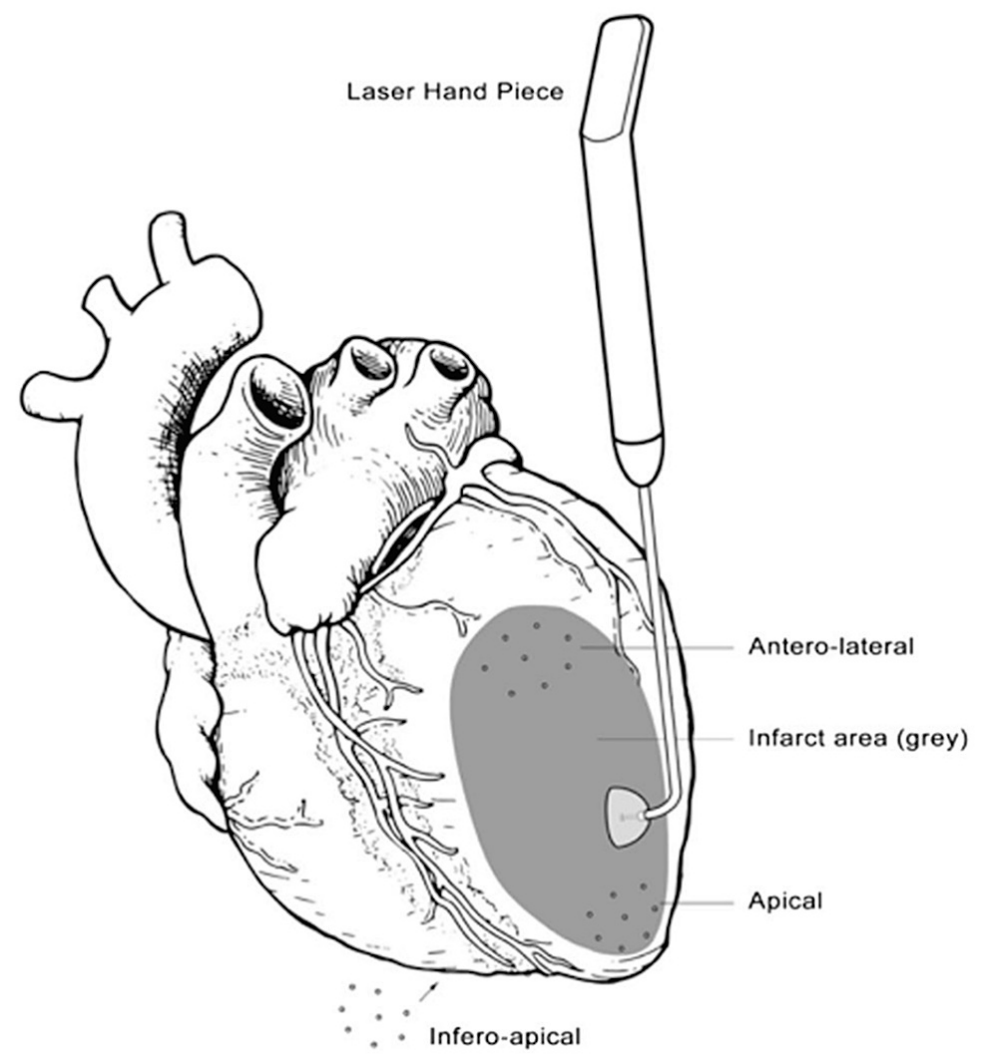
Transmyocardial revascularization using the handpiece. Transmyocardial revascularization (TMR) was performed on the heart by lasing channels in the myocardium, with an energy output of 7 W per laser pulse using the Ho:YAG fiber optic hand tool. The gray region seen on the heart represents an infarcted zone following ischemic damage. Depending on the ischemic region and the size of the patient’s heart, a total of 20–40 channels are created using the TMR laser handpiece. Typically, channels are placed on the anterolateral, apical, and inferoapical regions of the heart. The original figure was created by Dr. Zain Khalpey for a previous publication [19].

TMR therapy was performed concomitantly alongside CABG in this study. TMR involved the use of holmium:YAG laser (CryoLife, Kennesaw, GA) to create dozens of micropores in the myocardium. In this study, 10 channels were generated within the exterior left ventricle, depending on the ischemic region and the size of the patient’s heart. Channels were first employed on the inferior surface, moving towards the apex of the heart and subsequently on the lateral and anterior aspects of the epicardial surface (Figure 1) [26]. TMR channels were created on an arrested or beating heart depending on the type of laser being employed, CO_2_ or holmium:YAG. In our patients, those receiving TMR had 10 micropores (7 Watts/channel) created in the inferolateral wall of the left ventricle (LV) by Ho:YAG laser (CryoLife, Kennesaw, GA). Those receiving PRP had autologous PRP extract injected (1 ml, without calcium) around the TMR micropores, 5 mm adjacent. In patients treated with both TMR and PRP, 10 channels were made in the inferolateral LV with PRP extract injected in the peri-TMR zone, 5 mm adjacent to the channels.

Measurements

Demographic data, as well as data on comorbidities and clinical characteristics such as the Society of Thoracic Surgeons (STS) risk score and preoperative echocardiography findings (LVEF and GLS), were collected. Postoperatively, LVEF and GLS were measured using traditional echocardiography and speckle-tracking echocardiography (STE) at hospital discharge and at the one-year follow-up. These were our primary outcomes. Additionally, data on adverse events were recorded: postoperative atrial fibrillation (POAF), acute kidney injury (AKI), and the need for continuous renal replacement therapy (CRRT). AKI was defined using the Kidney Disease Improving Global Outcomes (KDIGO) criteria, which are internationally accepted and widely used [27]. POAF was defined using the criteria established by the STS for entry into the National Adult Cardiac Surgery Database [28]. These stipulate that the atrial fibrillation or flutter episode should last for at least 30 seconds. The episode should occur during the postoperative period, after cardiac surgery, and before hospital discharge. These criteria specify that atrial fibrillation or flutter should be documented by a 12-lead electrocardiogram (EKG), rhythm strip, or continuous telemetry monitoring. Patients with a history of preoperative atrial fibrillation or flutter were excluded unless it was successfully treated with ablation or surgery, and they had consistently been in normal sinus rhythm preoperatively. Lastly, reversible causes of atrial fibrillation, such as electrolyte imbalances or hyperthyroidism, should be ruled out or treated before making a diagnosis of postoperative atrial fibrillation.

Data collection and analysis

The results were compiled into a Microsoft Excel document (Microsoft Corporation, Redmond, Washington) on a secure server. Groups were stratified and compared in terms of their demographics, comorbidities, preoperative and postoperative measurements, as well as postoperative outcomes. T-tests and ANOVA analysis were used to determine significance, with a significance threshold of 0.05 as conventional. IRB approval and consent were obtained to interpret the data retrospectively (IRB#20200195).

## Results

**Figure 2:**
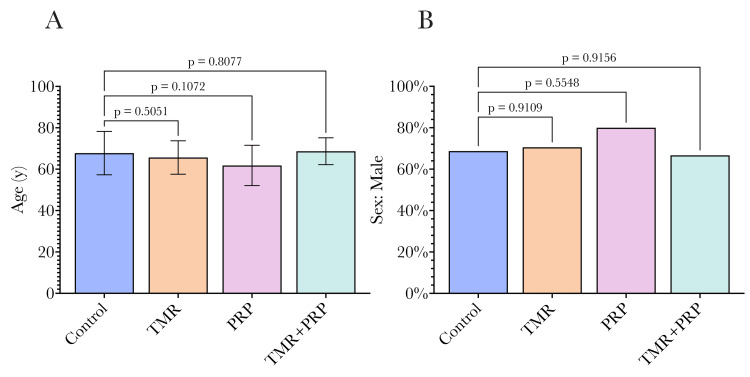
Demographic details of the four study groups. (A) Age; (B) male sex. No significant differences were found between study groups. Statistical comparisons were undertaken by comparing each group to the control group using ANOVA, with a p-value of 0.05 used as the significance threshold as conventional. TMR: transmyocardial revascularization, PRP: platelet-rich plasma.

Fifty-two patients (71% men, mean age 66 years) were enrolled in the study and randomized to four experimental groups: CABG only (n = 16); CABG/TMR (n = 17); CABG/PRP (n = 10); and CABG/TMR/PRP (n = 9). Demographic differences between groups are shown in Figure 2, with no significant differences between any of the four study groups.

**Figure 3:**
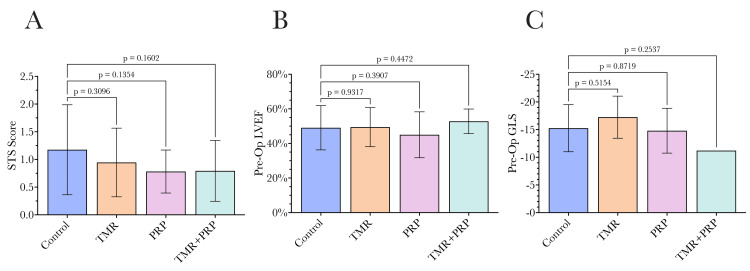
Clinical characteristics of the four study groups. (A) Society of Thoracic Surgeons (STS) risk score; (B) preoperative left ventricular ejection fraction (LVEF); (C) preoperative global longitudinal strain (GLS). All four groups were similar in terms of their STS risk score, preoperative LVEF, and preoperative GLS. Statistical comparisons were undertaken by comparing each group to the control group using ANOVA, with a p-value of 0.05 used as the significance threshold as conventional. TMR: transmyocardial revascularization, PRP: platelet-rich plasma.

Considering clinical characteristics, groups were similar in terms of STS risk score, preoperative LVEF, and GLS as shown in Figure 3.

**Figure 4:**
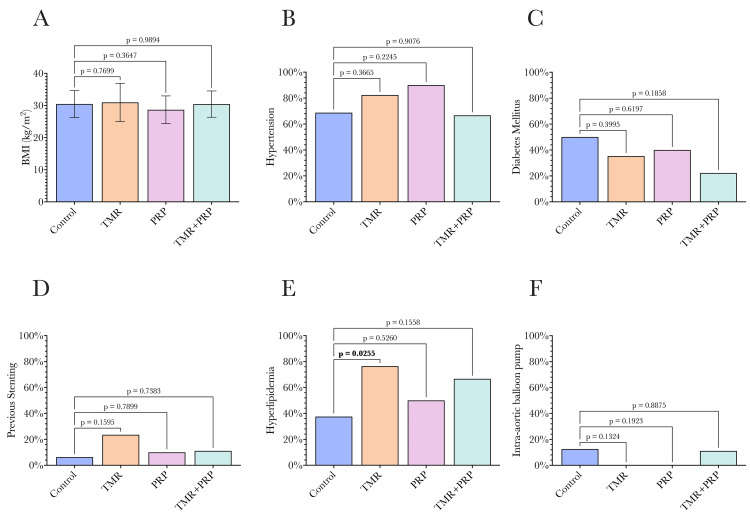
Comorbidities in each of the four study groups. (A) Body mass index; (B) hypertension; (C) diabetes mellitus; (D) previous stenting; (E) hyperlipidemia; (F) preoperative intra-aortic balloon pump use. All four groups were generally similar in terms of their comorbidities. The only significant difference was a significantly greater proportion of patients in the TMR-only group with hyperlipidemia compared to controls. Statistical comparisons were undertaken by comparing each group to the control group using ANOVA, with a p-value of 0.05 used as the significance threshold as conventional. BMI: body mass index, IABP: intra-aortic balloon pump, TMR: transmyocardial revascularization, PRP: platelet-rich plasma.

When preoperative comorbidities were considered, generally, groups were similar (Figure 4). However, the TMR-only group had a significantly greater proportion of patients with hyperlipidemia when compared to CABG controls (77% vs 38%, p = 0.02, Figure 4E).

 A full comparison of group demographics, as well as clinical characteristics and comorbidities, is shown in Table 1.

**Table 1:** Group demographics, clinical characteristics, and comorbidities. Demographics and preoperative characteristics of the patients in each of the four study groups. There was a significantly higher proportion of patients in the TMR-only group who had a history of hyperlipidemia. Otherwise, there were no significant differences between groups. *A significant (p < 0.05) different value compared to the control group, p-values calculated using ANOVA. Continuous variables are presented as mean ± standard deviation (SD), and categorical variables are presented as n (%). STS: Society of Thoracic Surgeons, LVEF: left ventricular ejection fraction, GLS: global longitudinal strain, BMI: body mass index.

Variable	Control, n = 16	TMR, n =17	PRP, n = 10	TMR+PRP, n = 9
Demographics				
Age, years, mean (± SD)	68 ± 10.5	66 ± 8.1	62 ± 9.7	69 ± 6.5
Sex, male	11 (69%)	12 (71%)	8 (80%)	6 (67%)
Clinical characteristics				
STS risk score, mean (± SD)	1.18 ± 0.81	0.95 ± 0.62	0.78 ± 0.39	0.79 ± 0.55
Preoperative LVEF	49.41 ± 11.30	45.00 ± 13.28	52.78 ± 7.12	49.06 ± 12.81
Preoperative GLS	-17.23 ± 3.80	-14.8 ± 4.05	-11.2	-15.25 ± 4.25
Comorbidities				
BMI, mean ± SD (kg/m^2^)	30.46 ± 4.21	30.96 ± 5.94	28.67 ± 4.32	30.43 ± 4.09
Hypertension	11 (69%)	14 (82%)	9 (90%)	6 (67%)
Hyperlipidemia	6 (38%)	13 (76%)*	5 (50%)	6 (67%)
Diabetes mellitus	8 (50%)	6 (35%)	4 (40%)	2 (22%)
Preoperative intra-aortic balloon pump use	2 (13%)	0 (0%)	0 (0%)	1 (11%)
Previous stenting	1 (6%)	4 (23%)	1 (10%)	1 (11%)

**Figure 5:**
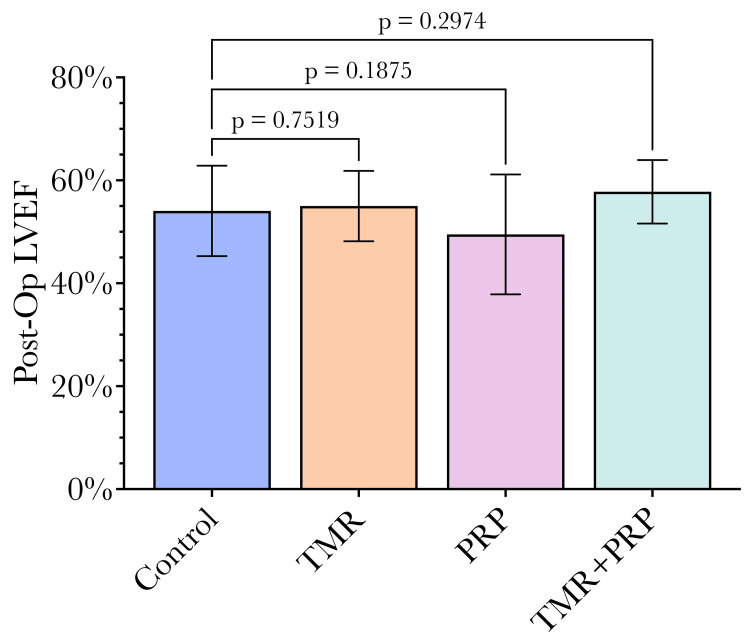
Postoperative LVEF was similar between groups. While differences in postoperative left ventricular ejection fraction (LVEF) between groups did not quite reach statistical significance, there was a trend toward a better LVEF in the TMR+PRP and TMR-only groups compared to the control. There was a trend towards a lower LVEF in the PRP-only group. Statistical comparisons were undertaken by comparing each group to the control group using ANOVA, with a p-value of 0.05 used as the significance threshold as conventional. TMR: transmyocardial revascularization, PRP: platelet-rich plasma.

Postoperatively, only LVEF was obtained as only standard echocardiography was undertaken. TMR/PRP patients had an increased mean EF compared to CABG alone (57.8% vs 54.1%, p = 0.24, Figure 5), though this did not quite reach statistical significance. Patients treated with TMR and PRP also had a greater immediate post-operative EF, although this did not quite reach statistical significance (57.8% ± 6.18) compared to TMR alone (55.0% ± 6.85) and PRP alone (49.5% ± 11.65). Patients were cared for post-operatively and discharged home in stable condition with plans to follow up in the outpatient clinic for re-evaluation. They would return to the office or hospital if their condition worsened.

**Figure 6:**
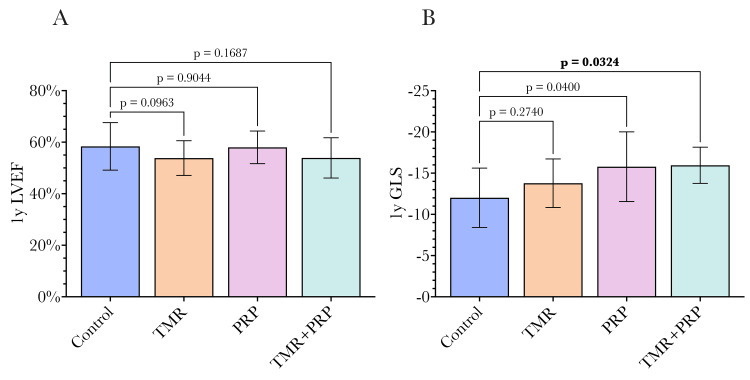
A significantly improved GLS was seen at one year in the TMR+PRP group. (A) Left ventricular ejection fraction (LVEF) at one year; (B) global longitudinal strain (GLS) at one year. There was a significantly improved GLS in the TMR+PRP group measured using speckle tracking echocardiography at one year postoperatively. Left ventricular ejection fraction (LVEF) at one year was similar between groups. Statistical comparisons were undertaken by comparing each group to the control group using ANOVA, with a p-value of 0.05 used as the significance threshold as conventional. TMR: transmyocardial revascularization, PRP: platelet-rich plasma.

In our study, we used one-year follow-up as the primary endpoint with measurements of GLS and EF at that time (Figure 6). At the one-year follow-up evaluation, patients treated with both TMR and PRP had a significant improvement in mean GLS compared to CABG alone (-15.96 ± 2.2 vs. -12.09 ± 3.34, p = 0.0163). Controls were also compared to the mean GLS of the TMR alone group (-13.78 ± 2.94, p = 0.2150) and PRP alone group (-15.79 ± 4.23, p = 0.1426), but the differences were not significant. Conversely, the mean left ventricular EF of the TMR/PRP group at the one-year follow-up was less than that of CABG control patients, though not significantly (53.89% ± 7.82 vs. 58.38% ± 9.22, p = 0.1687). The mean LVEF of the TMR alone group when compared to CABG controls was also less (53.82% ± 6.74 vs. 58.38% ± 9.22, p = 0.0963). The mean EF of the PRP alone group was most similar to that of the CABG controls (58.00% vs. 58.38%, p = 0.9044). A full comparison of primary outcomes is given in Table 2.

**Table 2:** Outcomes of interest in the four study groups. Study outcomes for the patients in each of the four study groups: postoperative left ventricular ejection fraction (LVEF) as well as LVEF and global longitudinal strain (GLS) at one-year follow-up. There was a significantly greater improvement in GLS in the TMR+PRP group. Otherwise, there were no significant differences between groups. *A significant (p < 0.05) different value compared to the control group; the p-values were calculated using ANOVA. All variables in this table are presented as mean ± standard deviation (SD). TMR: transmyocardial revascularization, PRP: platelet-rich plasma.

Variable, mean ± SD	Control, n = 16	TMR, n =17	p-value	PRP, n = 10	p-value	TMR+PRP, n = 9	p-value
Postoperative LVEF	54.06 ± 8.80	55.00 ± 6.85	0.7519	49.50 ± 11.65	0.1875	57.78 ± 6.18	0.2974
One-year follow-up LVEF	58.38 ± 9.22	53.82 ± 6.74	0.0963	58.00 ± 6.32	0.9044	53.89 ± 7.82	0.1687
One-year follow-up GLS	-12.09 ± 3.34	-13.78 ± 2.94	0.2150	-15.79 ± 4.23	0.1426	-15.96 ± 2.20*	0.0163

**Figure 7:**
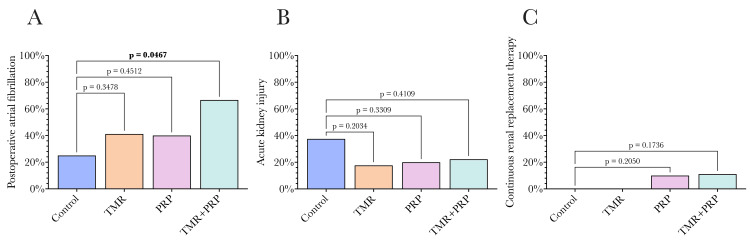
Adverse events across the four study groups. (A) Postoperative atrial fibrillation (POAF); (B) acute kidney injury as per KDIGO criteria [27]; (C) continuous renal replacement therapy. A significantly greater proportion of patients in the TMR+PRP group experienced POAF than the control group. Otherwise, groups were similar in terms of these adverse outcomes. Statistical comparisons were undertaken by comparing each group to the control group using ANOVA, with a p-value of 0.05 used as the significance threshold as conventional. KDIGO: Kidney Disease Improving Global Outcomes, TMR: transmyocardial revascularization, PRP: platelet-rich plasma.

Adverse events post-operatively were recorded for all groups with specific attention to POAF, AKI, and the need for CRRT, as shown in Figure 7. In terms of POAF, 25% (4/16) of CABG controls experienced POAF, while 67% (6/9) of TMR/PRP patients did, a significant difference (p = 0.0467). TMR alone and PRP alone also had an increased incidence of POAF, 41% (7/17) and 40% (4/10), respectively, compared to the control group, but neither demonstrated a significant difference (p = 0.3478 and p = 0.4512). In terms of AKI, 38% (6/16) of CABG controls experienced AKI, whereas 22% (2/9) of TMR/PRP patients did (p = 0.4109). TMR alone and PRP alone groups experienced AKI in 18% (3/17) and 20% (2/10) of patients, which was not significantly different compared to controls (p = 0.2034 and p = 0.3309). Only two patients required CRRT, one in the PRP alone group and one in the TMR/PRP group.

Of all patients enrolled in this study, only four were re-admitted throughout the first postoperative year; all four patients were part of the CABG-only control group. A full comparison of adverse events is shown in Table 3.

**Table 3:** Adverse events for the patients in each of the four study groups. There was a significantly higher proportion of patients in the TMR+PRP group who experienced postoperative atrial fibrillation. Otherwise, there were no significant differences between groups. *A significant (p < 0.05) different value compared to the control group; the p-values were calculated using ANOVA. Variables are presented as n (%). CRRT: continuous renal replacement therapy, TMR: transmyocardial revascularization, PRP: platelet-rich plasma.

Variable	Total, n = 52	Control, n = 16	TMR, n = 17	PRP, n = 10	TMR+PRP, n = 9
Postoperative atrial fibrillation	21 (40%)	4 (25%)	7 (41%)	4 (40%)	6 (67%)*
Acute kidney injury	13 (25%)	6 (38%)	3 (18%)	2 (20%)	2 (22%)
CRRT	2 (4%)	0 (0%)	0 (0%)	1 (10%)	1 (11%)
Re-admission <30days	0 (0%)	0 (0%)	0 (0%)	0 (0%)	0 (0%)
Re-admission >30 days	0 (0%)	0 (0%)	0 (0%)	0 (0%)	0 (0%)
Re-admission at one year	4 (8%)	4 (25%)	0 (0%)	0 (0%)	0 (0%)

## Discussion

Our study aimed to show that TMR combined with PRP as adjunctive therapy to CABG confers significant improvement in heart function over the year following the procedure.

We found that treating patients with TMR and PRP at the time of CABG provides a significant one-year improvement in cardiac function, as measured by GLS, in comparison to CABG alone. These data suggest a beneficial synergistic effect between TMR and PRP in the neovascularization and healing process prompted by TMR. Although past studies have shown mixed evidence for TMR efficacy in improving ejection fraction and overall heart function, we believe that this study serves as evidence that TMR and PRP are useful, synergistic adjunctive therapies to traditional revascularization with CABG, especially in diffusely diseased hearts.

Previous studies have also supported this theory, but our investigation with GLS provides further evidence. GLS has been reported to be a sensitive and accurate prognostic measurement in patients with heart failure. One large study (289 patients) found that those with lower absolute GLS values had increased mortality [29]. Additionally, a review of multiple large studies showed that absolute GLS predicts mortality in acute heart failure patients better than LVEF [30]. There are confounders with both measures in this study, as both EF and GLS can be user-dependent, but it seems that GLS may be less prone to this confounding factor. Importantly, GLS has been shown to be a more reproducible measure than EF, regardless of training [31]. Our study demonstrated a decreased GLS in patients who received TMR and PRP compared to CABG alone over the course of one year. These results signify an improvement in revascularization and outcomes.

We quantified the adverse events in all treatment and control groups, including AKI and POAF. Within the TMR/PRP treatment group, there was an increased incidence of POAF. This increased incidence could be indicative of the increased local inflammation in the myocardium induced by TMR. Additionally, the introduction of growth factors and inflammatory markers within the PRP could enhance the patient’s inflammatory response and contribute to the increased incidence of POAF. We hypothesize that this is further evidence of the inflammatory process that is the main driver of neovascularization and clinical benefits in patients receiving TMR.

There was no statistical difference in the incidence of AKI across the four treatment groups, which varied between 18% and 38%. Although AKI can be attributed to inflammation in some pathologic conditions, we do not believe that the increased local inflammatory response caused by TMR increases the risk of AKI in these patients. This is an encouraging sign that there is not a systemic inflammatory response to TMR or PRP in our study population.

Another adverse event was readmissions within a year following the operation. All readmissions within the study were seen in the control group (CABG alone). This could be attributed to persistent symptoms in these patients and lends further evidence to the reduced anginal symptoms seen in other studies of TMR patients. However, further investigation would be required as we did not record scores for angina symptoms. Our results are similar to other studies, which have reported that combination therapy of TMR and CABG for complete revascularization does not pose any increased mortality risk compared to CABG alone and is considered safe [9].

Limitations

This study has some limitations. In recent years, the transmyocardial laser revascularization probe is no longer manufactured by CryoLife, limiting the scope for replicability studies. However, cardiac shockwave therapy (SWT) works on similar principles of inducing small channels within the myocardium. SWT is a well‑established regenerative medical technique that is effective for the treatment of chronic tendonitis, long‑bone non‑union, and wound‑healing disorders. There has recently been increased interest in direct epicardial SWT devices, with the ongoing CAST-HF trial showing promising early results [32].

Patients and the surgeon were not blinded to the study, which could have influenced the patient’s perception of symptoms and the surgeon’s intraoperative decisions. While function was evaluated based on GLS and EF measurements, the study did not include patient-reported outcomes or specific questionnaires to evaluate symptom management of angina in the pre- and post-operative periods. Finally, patients were evaluated one year postoperatively, and this study does not measure outcomes past one year.

These outcome metrics highlight that a multifactorial approach to cardiac remodeling can improve heart function and even reduce the incidence of heart failure readmissions following surgery, as all readmissions in this study were from the CABG control group. To expand on these findings, it is important to note that only 10 micropores were made in a small portion of the myocardium. The number of pores could be increased to reasonably extend the improvements seen in this study. This study also introduces the need for preoperative stratification and planning to determine patients who would benefit from different adjunct therapies in addition to CABG. With further prospective trials and longer follow-up periods, concomitant adjunctive TMR with PRP extract therapy at the time of CABG may show durable and significant cardiac scar remodeling benefits, leading to heart recovery in CABG patients.

## Conclusions

In conclusion, our study demonstrates that the synergistic application of TMR and PRP as adjunctive therapies to CABG significantly improves cardiac function, as measured by GLS, one year postoperatively compared to CABG alone. This improvement in GLS suggests enhanced myocardial revascularization and remodeling. Despite an increased incidence of postoperative atrial fibrillation in the TMR/PRP group, potentially due to heightened local inflammation, no other adverse events were significantly different among the treatment groups. Further prospective trials with longer follow-up periods are warranted to confirm the durability and extent of these benefits.
